# Does Defensive Medicine Change the Behaviors of Vascular Surgeons? A Qualitative Review

**DOI:** 10.1155/2015/170692

**Published:** 2015-10-01

**Authors:** Paola Frati, Francesco Paolo Busardò, Pasqualino Sirignano, Matteo Gulino, Simona Zaami, Vittorio Fineschi

**Affiliations:** ^1^Department of Anatomical, Histological, Forensic and Orthopaedic Sciences, Sapienza University of Rome, Viale Regina Elena 336, 00161 Rome, Italy; ^2^Neuromed, Istituto Mediterraneo Neurologico (IRCCS), Via Atinense 18, Pozzilli, 86077 Isernia, Italy; ^3^Vascular and Endovascular Surgery Division, Department of Surgery “Paride Stefanini”, Policlinico Umberto I, Sapienza University of Rome, Viale del Policlinico 155, 00161 Rome, Italy; ^4^Department of Anatomical, Histological, Forensic and Orthopaedic Sciences, University of Rome Sapienza, 336 Viale Regina Elena, Lazio, 00161 Rome, Italy

## Abstract

Although in literature few successful claims have been shown in comparison with other medical specialties such as gynaecology and orthopaedics, vascular surgery is included among high-risk specialties. The high-risk of receiving medical claims may lead vascular surgeons to practice defensive medicine, as is normal in several other areas of clinical practice. No studies are available to our knowledge of the incidence of defensive medicine in the field of vascular surgery. Taking into consideration the scarce amount of information, the authors provide a critical discussion regarding the application of defensive medicine behaviour among vascular surgeons.

## 1. Introduction

Defensive medicine has been defined as the practice of ordering tests, procedures, and visits or the practice of avoiding treatments for patients considered at high-risk, in order to prevent medical malpractice claims [1, 2]. In the last decades, the culture of defensive medicine has grown worldwide because of the ever increasing number of medical claims, usually associated with high-risk medical areas [3]. As defensive medicine is used among physicians in order to lessen their exposure to medical malpractice litigation, this practice is constantly growing in all medical specialties on the presumption that every patient may be a potential litigant. In the course of time, defensive medicine has become a more and more transversal practice, encompassing all medical areas. A reduction of defensive medicine could help to improve the quality of medical care delivered by hospitals and in all medical specialties the diffusion of this phenomenon should be better controlled. Vascular surgery is a specialty of surgery in which diseases of the vascular system, or arteries and veins, are managed by medical therapy, minimally invasive catheter procedures, and surgical reconstruction. The specialty evolved from general and cardiac surgery as well as minimally invasive techniques pioneered by interventional radiology [1–3].

Although in literature a low incidence of successful claims has been shown in comparison with other medical specialties such as gynaecology and orthopaedics, vascular surgery is included among the high-risk specialties. A high-risk of receiving medical claims may lead vascular surgeons to practice defensive medicine, as in every other area of clinical practice. Nevertheless, to our knowledge, no studies are available in literature about the practice of defensive medicine in vascular surgery. The aim of this paper is to examine the defensive approach, not only in its current application, but also in the field of vascular surgery, discussing how being sued can affect medical treatment therefore highlighting the need to assess the real incidence of certain defensive behaviours among vascular surgeons.

## 2. Materials and Methods

The following databases (from 1978 to January 2015) Medline, Cochrane Central, Scopus, Web of Science, and Science Direct were used, searching with the following key words: vascular surgery, defensive medicine, medical negligence, and misdiagnosis. The main key word “vascular surgery” was searched for singularly and then associated individually with each of the other keywords.

Of the 446 sources found, only 22 were considered appropriate for the purpose of this paper. All sources have been screened independently by three physicians and in order to be included they had to be selected by at least two of them.

## 3. Some Data about Defensive Medicine

The phenomenon of defensive medicine adds 5% to 9% to the cost of US medical care and the annual medical liability system costs along with defensive practice have been estimated in 2008 as $55.6 billion of dollars equal to 2.4% of total health care spending [4, 5]. Recently, the Italian parliamentary board of inquiry estimated the cost of defensive medicine in the public sector at 10.5% while in the private sector it amounts to 14% of total health care spending. Therefore, according to the board, defensive medicine costs 10 billion euros, equal to 0.75% of the gross national product. Although several studies have been published about various approaches to defensive medicine, the consequences deriving from it are still controversial and debatable [6]. Tancredi and Barondess [2], for example, reported a comprehensive study supporting how defensive medicine does not determine the increase of health care costs but rather increases the exposure of patients toward unnecessary risks. In the same way, defensive medicine has been further defined as a widespread practice with little impact on medical care costs [7]. Defensive medicine, as reported by several clinical studies, has a negative impact on the quality of medical care, even without taking into consideration the price of this practice [8]. Several studies have highlighted how lawsuits have a negative impact on physicians causing them stress thereby jeopardizing their future performance [9]. The increase of public attention towards adverse events together with the patients' expectation in obtaining higher compensation for damage from medical malpractice has created a significant pressure on health professionals, making it more difficult for them to obtain adequate insurance coverage, particularly in some specialized branches (such as gynecology and orthopedics) more exposed to this risk. Medical claims are clearly based on the law of medical malpractice which still represents the main guarantee for patients and a useful tool for protecting the patients' health. Although medical liability discourages substandard care and allows patients to obtain reasonable damage compensation, there is no evidence in the literature that a fear of being sued is useful for reducing the rate of medical error [10]. In this perspective, the increase of exposure to medical malpractice has been making physicians more careful and conscious of their own actions. Considering a defensive approach we can identify two different forms: positive and negative. While the positive form can be defined as the practice of performing unnecessary therapeutic and diagnostic treatments, the negative form can be identified as the practice of declining to provide medical care. Both in positive and in negative form, the primary aim is to prevent medical claims, rather than to promote the patient's best interest [11].

## 4. Vascular Surgery between Defensive Medicine and Medical Malpractice

The exposure to medical malpractice litigation has also increased in the field of vascular surgery, where physicians are called to cure disorders affecting arteries, veins, and lymph vessels. As for every other area of clinical practice, vascular surgeons tend to be sued for failing to deliver safe and appropriate care to patients [12]. Physicians involved in vascular surgery may be sued when the execution of surgical treatments or preoperative activities (diagnostic procedures) are the result of medical malpractice. Because vascular surgeons are also frequently involved in other surgical treatments (elected for different reasons), due to intraoperative complications such as bleeding disorders, in examining the phenomenon of defensive medicine the general division between general and vascular surgery could be misleading. A study conducted by Campbell et al. [13] in England involving 424 claims revealed that varicose vein is the most common pathology involved in medical claims and nerve damage was the most frequent subject of complaints, followed by incorrect surgery and damage to the femoral vein and artery. Markides et al. in 2008 found that 50% of all successful claims were based on intraoperative problems, while 14% and 11% were assigned, respectively, to failure/delay of treatment and diagnosis. Varicose vein surgery was identified as the most common area of litigation, followed by peripheral vascular disease and abdominal aortic aneurysm. Both the studies above mentioned highlight the prevalence of varicose vein surgery in the management of medical claims regarding vascular surgery, taking into consideration that other diseases such as intraoperative nerve and vessel damage may cause worse permanent damage to the patient [14].

## 5. Discussion

The probability of defensive performances, among physicians, is directly proportional to the specific risk level. Among surgical specialties, vascular surgery is considered to be a high-risk of litigation as confirmed by Jena et al. [15] in a study of 2011, where the proportion of physicians dealing with malpractice claims has been evaluated taking into consideration each specialty. The results obtained show an almost 19% per year probability of facing a claim for vascular surgeons together with thoracic and cardiac surgeons [15]. Nevertheless, no study has yet been conducted in the literature about the incidence of defensive medicine in the specific field of vascular surgery. The exposure of vascular surgeons to medical malpractice litigation may be relevant both in emergency and in elective surgery, where the control of unexpected intraoperative bleeding may differently affect the physicians' performance. “Type of treatment” and “time factor” may also represent two important key points in the evaluation of defensive approaches among vascular surgeons. Reporting the main types of disease and procedures involved in successful medical claims recorded by the NHS Litigation Authority, Markides et al. highlighted the importance of a correct and appropriate treatment in the management of patients with peripheral vascular disease (PVD), abdominal aortic aneurysm (AAA), and carotid artery disease (CAD) [14]. The main issue involving vascular surgeons is due to the management of CAD and the probability of causing serious neurological damage (e.g., ictus) or, in the worst case, the death of the patient, posing an interesting question whether or not vascular surgeons should operate on patients with asymptomatic carotid stenosis. The proper treatment of carotid stenosis has always been of great interest for the vascular surgeons. In order to standardize the approach to this pathology, the Society for Vascular Surgery (SVS) published in 2008 specific guidelines for the treatment of carotid stenosis, providing recommendations on the basis of an evidence-based medicine approach [16, 17]. According to these guidelines, recommendations for medical therapy rather than surgical treatment depend on the grade of carotid stenosis, distinguishing among patients with a low, moderate, and severe grade of carotid stenosis. In this perspective, practical examples of positive defensive approaches with the aim of preventing medical claims in case of ictus or death should only be not following specific guidelines and performing surgical treatment without specific symptoms, together with an overestimation of diagnostic test results (e.g., Eco-Doppler), thereby exposing the patients to unnecessary surgery.

Further aspects should be considered in the occurrence of mistakes in surgical procedures. An interesting approach is the evaluation of “time factor” in vascular surgery, carried out by Sirignano et al, who highlighted, between 2009 and 2011, 63 claims involving vascular procedures and how the erroneous timing in surgical, medical, or diagnostic intervention may generate errors and how even more frequently errors occur in cases treated electively rather than in urgent or emergency cases [18].

An “emblematic” question may arise in the evaluation of erroneous timing in vascular surgery: which factors can affect the timing of elective procedures? In this perspective a useful support in the attempt to provide a valid answer is to underline the role of “defensive medicine,” which still remains controversial. A “positive” defensive medicine approach can occur in the assessment of “cardiac risk stratification” for vascular surgery, which is considered a very high-risk category, associated with cardiac morbidity rates greater than 5% in many reports. Examples include aortic and other major vascular surgeries, as well as peripheral vascular surgery [19].

For this reason, in order to avoid underestimation of the cardiac risk, vascular surgeons may assume an “assurance behavior,” which increases the time of preoperative investigation with economic repercussions.

With the aim of reducing this phenomenon, the guidelines of the American Association College of Cardiology and the American Heart Association recommend preoperative cardiac testing only when the results may influence patients' management. Moreover, they individuated four high-risk conditions, showing how to assess and treat them preoperatively; they include unstable coronary syndromes, decompensated heart failure, significant cardiac arrhythmias, and severe valvular disease. Applying these guidelines in an appropriate manner could help in reducing the risks of incurring in medical defensive procedures.

If, on the one hand, the application of positive defense medicine in vascular surgery can impact on the consequences above reported, on the other hand, the effects of negative defense medicine can be even more dangerous, as it is based on “*avoidance behaviors*” only with the aim of reducing the risk of litigation in certain medical activities. However, according to data reported below, this attitude may sometimes increase the number of claims. The authors of the same study [18] have also tried to evaluate the “time factor” and how it can affect the final outcome in patient treatment.

In a paper of 2012 [18] five cases of claims in vascular surgery, in which the “time factor” played a key role in delaying or in not performing a surgical procedure, were reported. In one case, the delay in performing the ligation of an arteriovenous fistula in the left arm of a 75-year-old woman undergoing haemodialysis, which was diagnosed in September and not operated on until the next December due to numerous follow-up visits, caused a severe bleeding in the arm and the death of the patient shortly after. Another case involved a 63-year-old male, who after an intervention of saphenectomy complained of a severe pain in his foot, which was not treated causing, on the 4th postoperative day, the amputation of the leg, for a severe ischemia of the foot due to a thrombosis of popliteal and tibial arteries. Because amputation of a limb is a drastic solution, intolerable for a patient to take into consideration even if suffering from critic ischemia, a therapeutic approach based on an excessive use of revascularization therapies aiming only to obtain a defense in a potential lawsuit is another pragmatic example of positive defensive medicine.

Other important issues worthy of attention, which should be evaluated, are the different medico-legal implications of endovascular versus open surgery and the peculiar features of vascular surgery from the perspective of defensive medicine and the consequent medico-legal problems that may result. Regarding the first aspect, over the past 30 years, vascular surgery has undergone a significant evolution process: all has changed with the advent of endovascular procedures and, thanks to the rapid evolution of available grafts, everything is still changing. The approach to patients has varied as well as indications. Critically impaired patients, in poor general condition, can be subjected to effective treatments that were not first to be proposed for the high-risks associated with open surgery [20]. The availability of those new “less invasive” endovascular procedures could have influenced some “inexperienced” surgeons of the existence of an easier way to treat patients. However, this assumption has to be considered wrong. The new endovascular procedures represent, together, a very efficient weapon for the clinician but for an experienced clinician, at the end of an adequate learning curve. A modern vascular surgeon must be a surgeon, capable of working with the same skills in both open and endovascular surgical procedures [21].

Finally, in considering the fields of application of vascular surgery from the perspective of defensive medicine and the consequent medico-legal problems that may result, three key aspects have to be carefully evaluated: the indication, the timing, and the technique used; in Table 1 are reported four pathologies of vascular surgery: carotid stenosis, aortic aneurysms, aortic dissections, and peripheral arterial disease, which are evaluated in the light of the three indicators above mentioned [20, 21].

## 6. Conclusions

In conclusion, the little literature available about defensive medicine related to vascular surgery leads us to take seriously into consideration the need to assess the real incidence of defensive medicine in this area. In order to assess the current trend of defensive medicine (positive and negative) in vascular surgery, we suggest focusing not only on the personal perceptions of vascular surgeons practicing it, which however could be underestimated taking into account “*avoidance behaviors*,” but also on reporting and reevaluating any suspicious case with a team of specialist surgeons, who can evaluate all diagnostic and surgical procedures undertaken together with the compliance to the guidelines and runtimes. Only in this way will it be possible to have a true picture of this phenomenon and therefore be able to correct the negative impact it has in terms of cost and quality on the health service.

## Figures and Tables

**Table 1:** Vascular surgery pathologies: carotid stenosis, aortic aneurysms, aortic dissections, and peripheral arterial disease, evaluated in the light of the indication, timing, and technique.

	Indication	Timing	Techniques
Carotid stenosis	To treat symptomatic stenosis or only haemodynamic asymptomatic lesions	Within 24 hours from symptoms onset or within 14 days	Carotid endarterectomy or carotid artery stenting

Aortic aneurysms	To treat on the basis of the diameter or on the basis of accompanying symptoms and/or aortic morphology	In case of symptoms: as soon as possible or after careful patient evaluation	Open repair or endovascular repair

Aortic dissections	To treat all the dissection or selectively on the basis of visceral malperfusion, aneurismatic dilatation, and uncontrolled pain and hypertension	Within 15 days from symptoms onset or after 15 days	Open repair or endovascular repair

Peripheral arterial disease	To treat patients presenting with claudication or only patients with critical limb ischemia	Critical limb ischemia presenting patient should always be treated in urgent/emergent setting	Open repair or endovascular repair. In case of endovascular repair, use or do not use stent, covered stent, and drug eluting devices
	To recognize patients presenting with acute limb ischemia		
